# An enhanced participant information leaflet and multimedia intervention to improve the quality of informed consent to a randomised clinical trial enrolling people living with HIV and obesity: a protocol for a Study Within A Trial (SWAT)

**DOI:** 10.1186/s13063-021-05979-y

**Published:** 2022-01-17

**Authors:** Lydia O’Sullivan, Stefano Savinelli, Stephen O’Hare, Sinéad Holden, Ciara McHugh, Patrick Mallon, Peter Doran

**Affiliations:** 1 School of Medicine, University College Dublin, Dublin 4, Ireland; 2 Health Research Board-Trials Methodology Research Network, Galway, Ireland; 3 Saint Vincent’s University Hospital, Elm Park, Dublin 4, Ireland; 4 Centre for Experimental Pathogen Host Research, University College Dublin, Dublin 4, Ireland; 5 HIV Ireland, Eccles Street, Dublin 7, Ireland; 6 Clinical Research Centre, Mater Misericordiae University Hospital, School of Medicine, University College Dublin, Dublin 4, Ireland

**Keywords:** Informed consent, Clinical trials, Study Within A Trial (SWAT), Patient and public involvement (PPI), Multimedia, Participant information leaflets

## Abstract

**Background:**

It is the investigator’s responsibility to communicate the relevant information about a clinical trial to participants before they provide informed consent to take part. Systematic reviews indicate that participants often have a poor understanding of the concepts which are key to ensuring valid informed consent, such as randomisation and risks/discomforts. Paper-based participant information leaflets and informed consent forms (PIL/ICFs) are becoming longer and are often too complex for many participants. Multimedia interventions and enhanced PIL/ICFs have been trialled in an attempt to improve participants’ understanding of various aspects of research studies. However, there is insufficient empirical evidence to determine how effective such interventions are. This protocol describes a study to evaluate whether an enhanced PIL/ICF and website help research participants to understand important information about a human immunodeficiency virus (HIV) randomised clinical trial.

**Methods:**

This Study Within A Trial (SWAT) is a prospective, multi-centre, randomised, controlled, parallel-group study embedded in a host clinical trial. The host trial (the SWIFT trial; EudraCT: 2019-002314-39) is a prospective, multi-centre, randomised, open-label, controlled trial investigating if semaglutide along with dietary advice assists individuals with HIV and obesity to lose weight, compared to dietary advice alone. For the SWAT, participants will be randomised in a 1:1 ratio to either the control (standard PIL/ICF) or the intervention (an enhanced PIL/ICF and a website which includes animations). The enhanced PIL/ICF and website were developed in line with the guidance from organisations which promote plain English and accessible public-facing materials in conjunction with HIV Ireland, a HIV advocacy organisation, and our previous work on consent documents. The primary outcome of the SWAT is the quality of informed consent, assessed by a validated comprehension test—the modified Deaconess Informed Consent Comprehension Test (DICCT). The DICCT will be administered within 48 h of consent to the host trial. The secondary is recall, measured by the modified DICCT questionnaire scores 2 weeks post-consent to the host trial.

**Discussion:**

The results of this SWAT will add to the methodological evidence base on the use of multimedia to improve the quality of informed consent to randomised clinical trials.

**Trial registration:**

ClinicalTrials.govNCT04174755. EudraCT 2019-002314-39. SWAT 160, Northern Ireland Hub for Trials Methodology Research SWAT repository (Clarke M, et al., Trials. 16:P209, 2015).

**Supplementary Information:**

The online version contains supplementary material available at 10.1186/s13063-021-05979-y.

## Background

Informed consent depends on the communication of relevant information, capacity to consent, and voluntariness [1]. The provision of accurate, useful, and understandable information is therefore an important part of the informed consent process, to preserve the autonomy of the research participant and protect their rights [2]. The Good Clinical Practice (GCP) guidelines outline the information which should be given to research participants, including, among others, the aim of the trial; the nature of the trial treatments including randomisation, where applicable; and any risks or inconveniences to the participant [3]. By providing information, explaining the study, and responding to questions and concerns, the research staff ensure the integrity of the consent obtained. It is the investigator’s responsibility to ensure participants have all the required information before they give their consent to take part in a clinical trial [1]. However, there is an increasing body of evidence, causing significant concern, over the true level of understanding of research participants. Recent systematic reviews have suggested that research study participants often have a poor understanding of vital concepts which are important in ensuring valid informed consent [4, 5]. This includes concepts surrounding randomisation and the risks/side effects of participating, which participants find particularly difficult [6–10]. This lack of understanding impairs research participants’ ability to make informed choices, undermining their autonomy. While research participants often express good levels of satisfaction with the information they are provided with, Bertoli and colleagues’ survey of 114 participants in arthritis trials demonstrated that satisfaction did not correlate with objective understanding [7].

In addition to the discussions with the research team, the primary route through which information is conveyed to participants is the paper-based participant information leaflet (PIL), a document detailing the information about the study including study objectives, design, procedures, and information on insurance and data protection. The efficacy of the PIL as an effective means of conveying complex information is receiving significant attention within clinical trials settings. Recent studies by our group and others have suggested that these documents are becoming longer [11] and are often too complex for many participants [12, 13]. Multimedia and digital interventions, such as websites, videos, and computer presentations, have been tested to determine whether they improve participants’ understanding and the rate of recruitment [14, 15]. However, despite some initially promising results, there is insufficient empirical evidence on how effective they are at improving participants’ understanding of the research and implications of participating [15–17]. There is also limited evidence that using a multimedia intervention can improve recall at a later time point [18, 19]. Similarly, the results of the studies which explored the use of an enhanced PIL/ICF are difficult to generalise from as some were simulated rather than real-life consent scenarios [20–22] and samples with varying literacy rates were used [23–26]. It is important that interventions to strengthen the methodology of clinical trials are tested within actual trial settings, rather than in simulated or hypothetical situations [27]. A Study Within A Trial (SWAT) aims to add to the methodological evidence base by embedding a study within a ‘host trial’, without interfering with the conduct or outcomes of that host trial [28]. Evidence to improve the informed consent process by enhancing the presentation of information should ideally be conducted as a SWAT. This paper describes a protocol for a study which aims to evaluate whether a multimedia resource, provided in addition to an enhanced PIL/ICF helps research participants to understand important information about a human immunodeficiency virus (HIV) clinical trial.

## Methods

This SWAT protocol is written in accordance with the guidelines for reporting embedded recruitment trials described by Madurasinghe on behalf of the Medical Research Council Systematic Techniques for Assisting Recruitment to Trials (MRC START) Group [27]. The completed reporting checklist produced by this group, which was adapted from the Consolidated Standards for Reporting Trials (CONSORT) 2010 statement, is included as a supplementary file (see Additional file 1). This SWAT has been registered with the Northern Ireland Hub for Trials Methodology Research SWAT repository (SWAT Number #160) [29].

### Objectives

The primary objective of this SWAT is to determine if the use of a multimedia intervention and enhanced PIL/ICF improves the quality of informed consent, measured within 48 h of the provision of written informed consent to participate in a host clinical trial.

The secondary objective is to determine if a multimedia intervention and enhanced PIL/ICF improve the recall of information relevant to informed consent 2 weeks after the provision of written informed consent.

### Study design

**Fig. 1:**
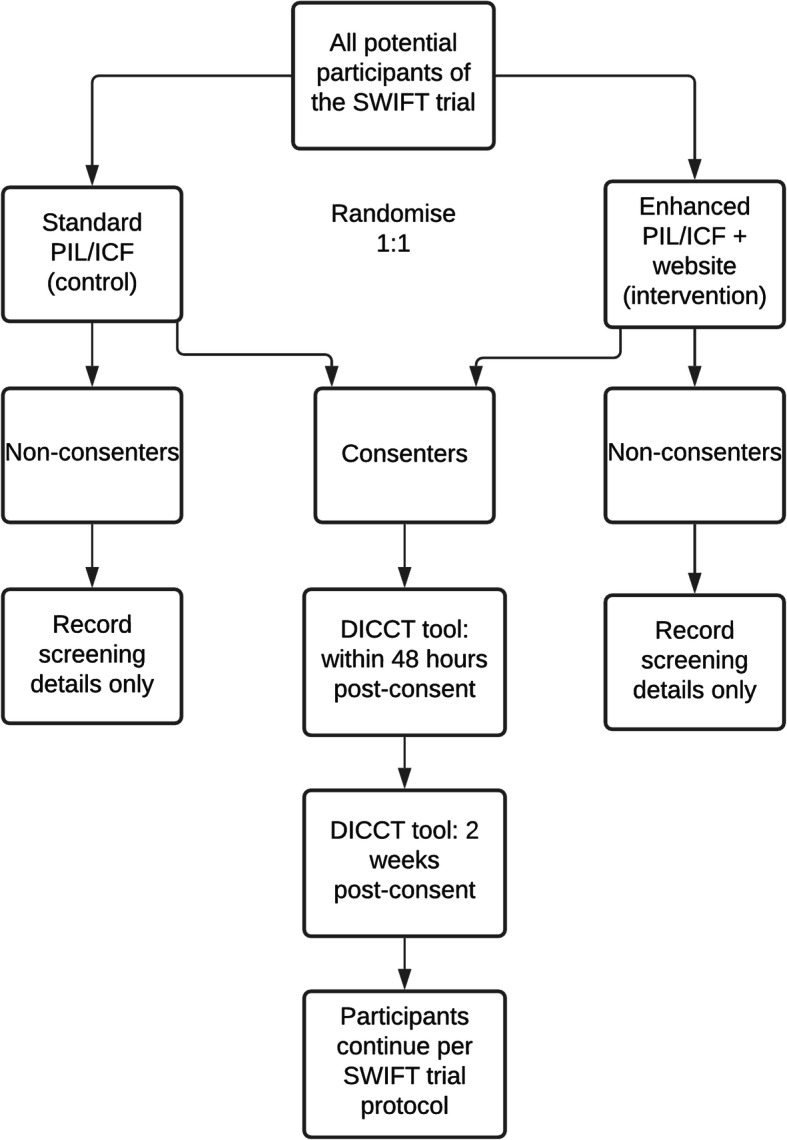
Design of this Study Within A Trial (SWAT) using the SWIFT trial as the ‘host trial’. DICCT, Deaconess Informed Consent Comprehension Test; PIL/ICF, participant information leaflet/informed consent form; SWAT, Study Within A Trial; SWIFT Trial, the host trial for this SWAT)

This is a prospective, multi-centre, randomised, controlled, parallel-group study embedded in a host trial following the Study Within A Trial (SWAT) methodology [28]. Figure 1 describes the design of the SWAT.

### Study setting

The host trial (the SWIFT trial; EudraCT No: 2019-002314-39) is a prospective, multi-centre, randomised, open-label, controlled trial investigating if semaglutide helps people living with HIV and obesity to lose weight [30]. Eighty participants will be randomised onto the SWIFT trial in Ireland and Denmark. The primary endpoint of the SWIFT trial is the change in total body weight at week 28. Secondary endpoints of the SWIFT trial include change from baseline to week 28 for the following variables: total and subcutaneous fat, markers of B cell and T cell function, markers of innate immunity, inflammation, gut microbiome composition, adipose tissue function, lipid profiles and glucose metabolism, HIV RNA and HIV viral reservoir, health-related quality of life, and the proportion of participants in both arms not achieving ≥ 5% weight loss at week 16. The SWAT will take place at the two Irish recruiting sites for the SWIFT trial (the host trial)—the Mater Misericordiae University Hospital and Saint Vincent’s University Hospital, both located in Dublin, Ireland. Participants will provide written informed consent to take part in the SWAT at the same time as the SWIFT trial (the host trial). The informed consent process will be facilitated by the SWIFT research team and will be conducted in compliance with the Declaration of Helsinki [2] and Good Clinical Practice guidelines [3].

### Eligibility criteria

Informed consent to take part in the SWAT will be included in the informed consent process for the SWIFT trial (the host trial). The key inclusion criteria for the SWIFT trial include the following:

- Adults ≥ 18 years who are HIV-positive
- On stable HIV treatment with a suppressed viral load for the previous 2 years
- A CD4 white cell count > 200 cells/μL for at least 1 year
- Body mass index (BMI) of 30 kg/m^2^ or higher or BMI between 27 and 30 kg/m^2^ and high blood pressure and/or diabetes and/or high cholesterol

The key exclusion criteria for the SWIFT trial include the following:

- Severe renal or liver impairment
- Obesity induced by other endocrine disorders or the use of anti-psychotic medications associated with weight gain
- Cancer (apart from treated Kaposi’s sarcoma)
- Users of illicit intravenous drugs
- Enrolment in another clinical trial of an investigational medicinal product
- Pregnancy or breastfeeding
- Inability to self-administer subcutaneous semaglutide

There are no additional inclusion/exclusion criteria for the SWAT.

### Description of study intervention and control and their allocation

The control in this SWAT is a standard PIL/ICF which has received Research Ethics Committee (REC) approval. The intervention in this SWAT is an enhanced paper PIL/ICF designed to improve readability and understandability and a website, co-designed by a patient and public representative from HIV Ireland (a HIV advocacy group), the SWIFT research team, and the SWAT research team. The materials in the intervention arm have also received REC approval. While the informational content in both control and intervention arms is the same, the enhanced PIL/ICF and website were written and prepared by a professional graphic designer with the aim of adhering to guidelines produced by literacy agencies for public-facing documents, to maximise accessibility [31–33]. This approach included, among other factors, the following: using the active voice wherever possible, keeping the average sentence length between 15 and 20 words, using infographics and images, using a question/answer format to encourage processing of information, using simple or commonly used language (Plain English) wherever possible, and explaining unfamiliar or technical terms. The website and the enhanced PIL/ICF contain the same informational content, but the website also includes some animations which support the written content. The animations on the website were developed in conjunction with the Educational Technology team at the School of Medicine, University College Dublin, and focus mainly on two aspects which have been demonstrated to be poorly understood by research participants: randomisation and risks/side effects [5]. The enhanced PIL/ICF is included as a supplementary file (see Additional file 2). The website can be viewed here https://swifttrial.ucd.ie.

**Fig. 2:**
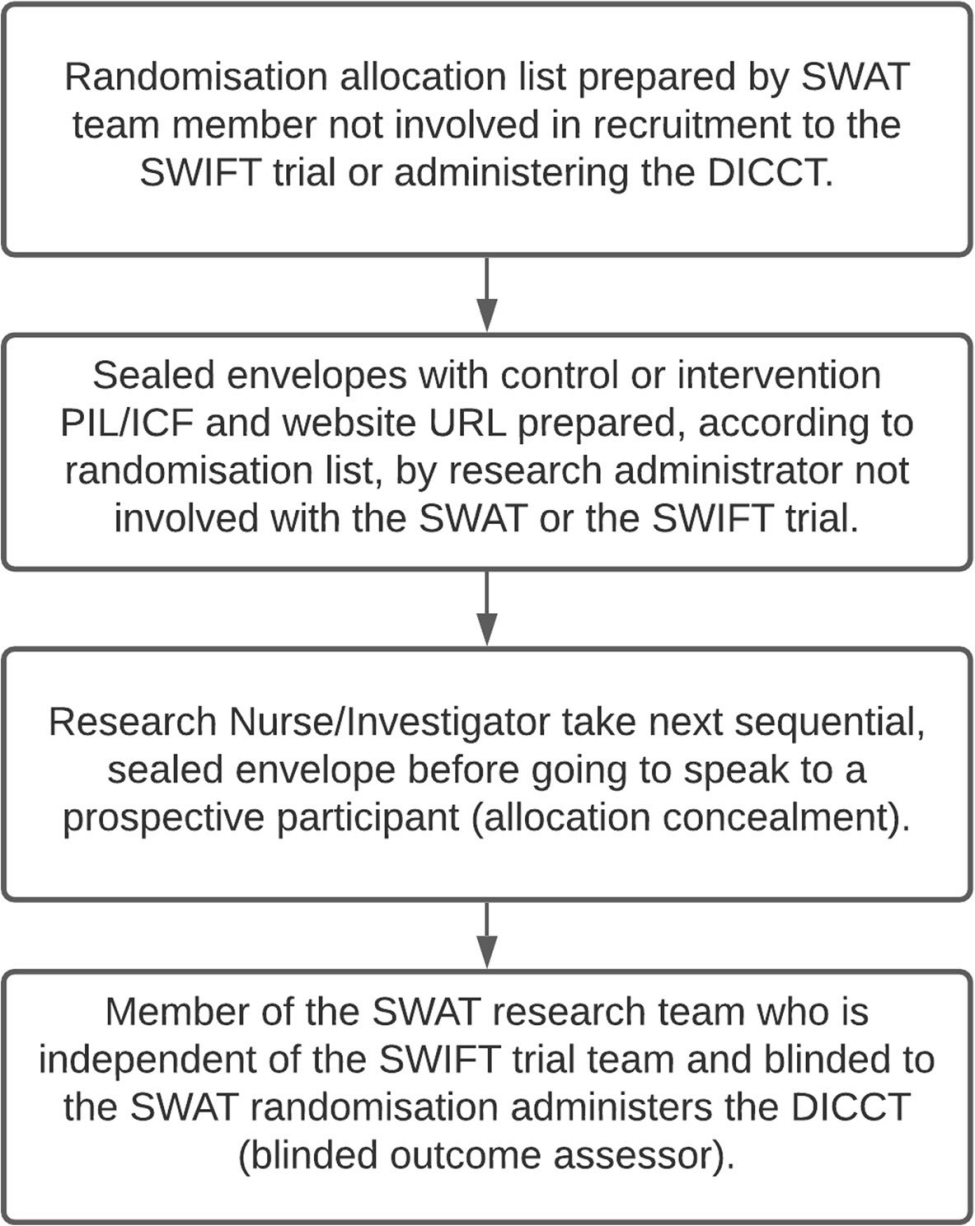
Schematic describing the preparation of recruitment pack, allocation concealment, and blinding. DICCT, Deaconess Informed Consent Comprehension Test; PIL/ICF, participant information leaflet/informed consent form; SWAT, Study Within A Trial; SWIFT Trial, the host trial for this SWAT)

Participants will be randomised in a 1:1 ratio between the control arm (standard PIL/ICF) and the intervention (enhanced PIL/ICF and access to the trial website) using blocks of six. A computer-generated randomisation allocation list has been prepared by a member of the SWAT research team who is not involved in recruitment to the SWIFT trial (the host trial) or in the assessment of the SWAT outcomes (SH). Sealed envelopes have been prepared by a research administrator who is not involved in the SWAT or the SWIFT trial, according to the allocation list (i.e., with either a standard or enhanced PIL and URL for the website). The clinical research nurse or investigator at each of the two participating sites will take the next consecutive sealed envelope before going to speak to a prospective participant for the SWIFT trial (the host trial), so the randomisation allocation for the SWAT will be concealed to reduce bias. To ensure that the research participants assigned to the intervention arm (enhanced PIL/ICF and website) access the website, tablet computers will be available in the research clinics. Following input from our public-patient partner at HIV Ireland, the website has also been designed to be accessible on smartphone devices used by many potential research participants as their sole means of accessing the internet. The outcome assessor for the SWAT (LOS) will be blinded to the participant randomisation for the SWAT. Figure 2 summarises this process.

### Outcomes

The primary outcome is the quality of consent, as measured by the modified Deaconess Informed Consent Comprehension Test (DICCT) questionnaire scores, assessed 48 h post-consent to the SWIFT trial (the host trial)—this is a continuous variable. The secondary outcome is recall, as measured by the modified DICCT questionnaire scores 2 weeks post-consent to the SWIFT trial (the host trial). The development and validation of the DICCT tool is described in full by Miller et al [34]. In brief, the tool consists of 14 questions, all of which have equal weighting and were written at an eighth-grade level (reading age of ~13 years). Each question is scored as follows: two points (correct answer), one point (correct but incomplete answer), or zero points (incorrect answer or no answer). Therefore, the maximum possible score is 28. The inter-rater reliability of the tool was found to be excellent. The following small modifications were made to the tool for this SWAT: the name of the hospital was changed so that the name of the host hospitals was used instead, and the word ‘subject’ was changed to ‘participant’. The DICCT assessment will be administered either face to face in clinic with participants or by phone. The primary and secondary outcomes will be assessed by one researcher (LOS) who will not be involved in the recruitment for the SWIFT trial and who will be blinded to the participant randomisation. The following variables will also be collected: rate of recruitment to the SWIFT trial (the host trial) across both arms of the SWAT, educational level of participants, and ethnicity of participants.

### Participant timeline

It is anticipated that recruitment to this SWAT will take place between July and December 2021, depending on the recruitment to the SWIFT trial (the host trial). There will be two assessment points in this SWAT: within 48 h and 2 weeks post-consent to the SWIFT trial.

### Data collection and management

Collected data will be inputted and stored in an Excel database held securely on one researcher’s password-protected laptop. The data (DICCT scores) collected for this SWAT will be coded using the participant’s screening number for the SWIFT trial. There will be no requirement to store personal data for the purposes of the SWAT analysis.

### Sample size

The target sample size for the SWIFT trial (the host trial) is 80 participants. Since this is a SWAT, the sample size will be decided by the SWIFT trial (the host trial), and no formal sample size calculation was performed. However, it is estimated that a difference of 15% (an effect size of 0.76) could be detected in DICCT scores with 80% power and a two-sided alpha of 0.05 with a sample size of 28 in each group. It is hoped that the results of this SWAT will ultimately be combined with the results from other similar SWAT in a meta-analysis.

### Statistical methods

Statistical analysis will be performed using the Statistical Package for the Social Sciences (SPSS) (IBM SPSS Statistics for Windows, version 24.0. Armonk, NY: IBM Corp.). Descriptive statistics will be used to outline the educational level and ethnicity of participants and the rate of recruitment to the SWIFT trial (the host trial) across the two arms of the SWAT. Depending on the distribution of data gained from the primary outcome of the SWAT, the appropriate test (parametric or non-parametric) will be selected to compare the DICCT scores in the two arms of the SWAT: an independent samples *t*-test or a Mann-Whitney *U* test.

## Discussion

The generalisability of SWATs is often limited by their sample size; however, the results of this SWAT will add to the growing body of evidence about whether multimedia resources and enhanced PILs/ICFs help research participants understand key information before they make a decision whether to take part or not. As noted above, an advantage of the SWAT methodology is that the investigation is conducted in a real trial recruitment setting, rather than a simulation involving participants making a hypothetical decision, which increases the reliability of the results. A key strength of this SWAT is that the enhanced PIL/ICF and multimedia intervention in this SWAT were co-produced with a representative from HIV Ireland, a HIV advocacy group—this ensures that the enhanced PIL/ICF and website are acceptable and intelligible to the target population for the SWIFT trial.

## Limitations

The unique context of this SWAT may limit the generalisability of the results.

## Trial status

At the time of submission of this article, participant recruitment to this SWAT had not begun. It is estimated that the recruitment to the SWAT will commence in August 2021 and will be completed by December 2021. The SWAT protocol number is V1.0/6-Aug-2021.

## Supplementary Information

**Additional file 1.** CONSORT 2010 checklist of information to include when reporting a randomised trial.

**Additional file 2.** Enhanced PIL/ICF for the SWIFT Trial.

## Data Availability

At the end of the study, data not already made publicly available in the publication of the SWAT results will be provided by the corresponding author on reasonable request.

## Abbreviations

HIV: Human immunodeficiency virus
MRC: Medical Research Council
ICF: Informed consent form
PIL: Participant information leaflet
REC: Research Ethics Committee
START: Systematic Techniques for Assisting Recruitment to Trials
SWAT: Study Within A Trial
SWIFT trial: This is the host trial for this SWAT (Trial name: Semaglutide’s Efficacy in Achieving Weight Loss for Those With HIV)
