# Supplementary material for: An enhanced participant information leaflet and multimedia intervention to improve the quality of informed consent to a randomised clinical trial enrolling people living with HIV and obesity: a protocol for a Study Within A Trial (SWAT)
Source: Trials. 2022 Jan 17;23:50. doi: 10.1186/s13063-021-05979-y (PMC8762861; doi:10.1186/s13063-021-05979-y)
Supplement: Supplementary file 2 — Additional file 2. Enhanced PIL/ICF for the SWIFT Trial. [file 13063_2021_5979_MOESM2_ESM.pdf]

THE MATER HOSPITAL

Ospideal Ollscoile Mater Misericordiae

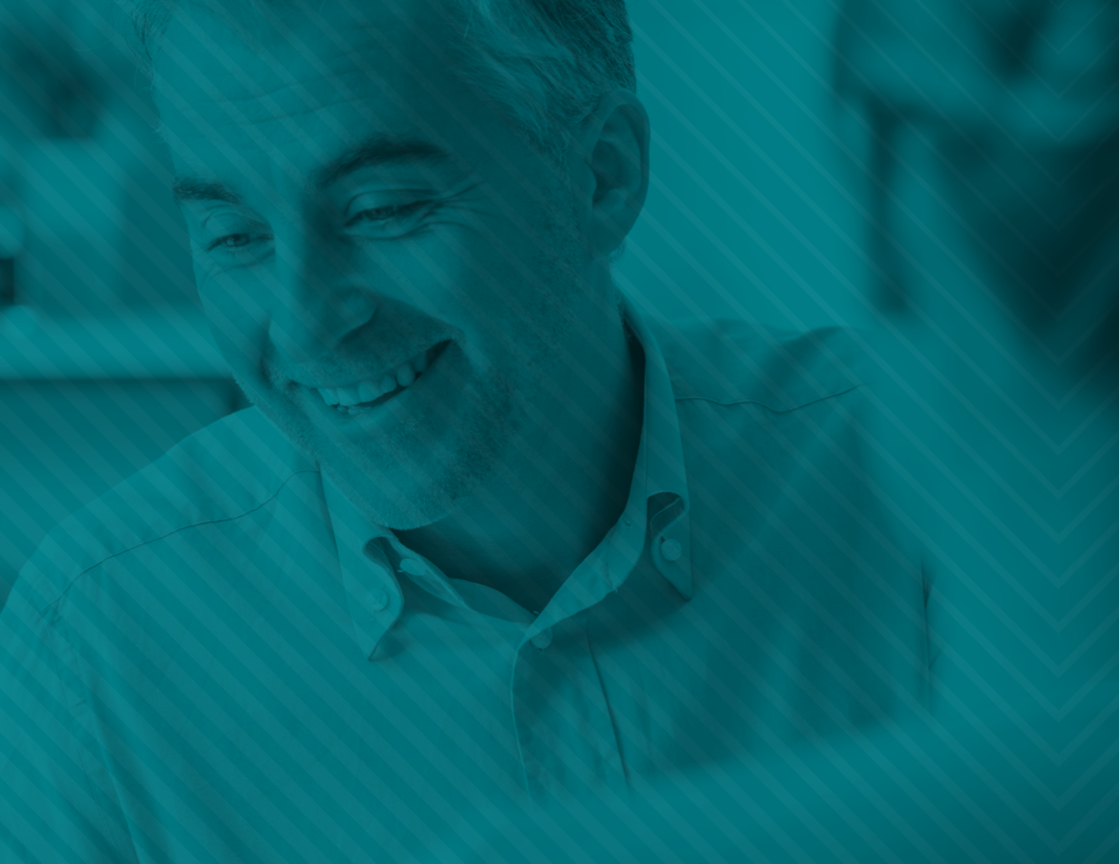

Semaglutide's Efficacy in Achieving Weight Loss for those Living with HIV
SWIFT Study

Patient Information

Protocol Number: 'Semaglutide_2019'

Lead study doctors:

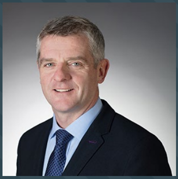

Prof. Patrick Mallon
St Vincent's University Hospital

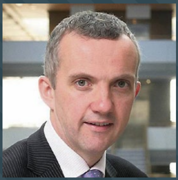

Prof. Donal O'Shea
St Vincent's University Hospital

Lead study doctor at the Mater Misericordiae University Hospital:

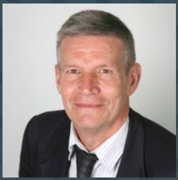

Prof. Jack Lambert

Welcome.

We invite you to take part in a research study for people with HIV and obesity. Before you decide if you would like to take part, please read this information leaflet carefully and ask any questions you might have.

Thank you for taking the time to read this leaflet.

Paddy Mallon

Prof. Patrick Mallon
St Vincent's University Hospital

Contact details for study doctor

» Tel: (01) 716 5821 (office hours)

Contents

What is the aim of this study? 5

What will happen if I agree to take part? 7

What will happen at the study visits? 10

Are there any benefits to taking part? 13

Are there any risks in taking part? 14

Questions you may have 17

Data Protection and Confidentiality 19

Consent Form 20

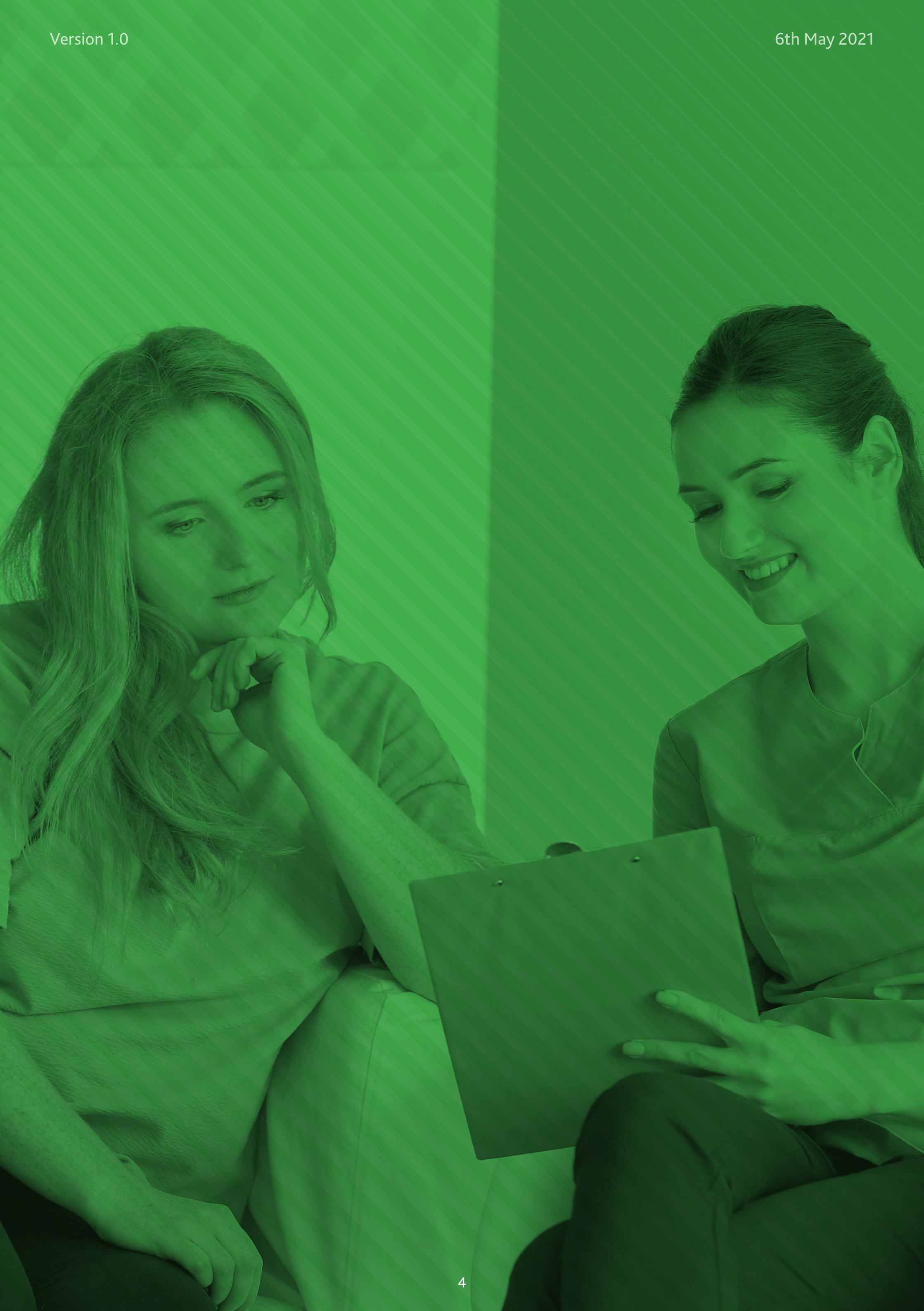

What is the aim of this study?

The main aim of this investigational study

To test to see if a drug called semaglutide helps 80 people living with HIV and obesity to lose weight

In addition, our study will examine if semaglutide changes the bacteria in the gut. We will also examine whether semaglutide changes the level of HIV in the immune system cells.

Semaglutide is a new treatment, already approved for managing blood sugars in diabetes. Early studies have also shown that it might help people with obesity to lose weight.

Q: Why have I been asked to take part?

We are asking you to take part because you are living with HIV and have a body mass index of 30kg/m^2.

Q: Do I have to take part?

No. You do not have to take part. Your decision will not change your medical care now or in the future. If you decide to take part, you can change your mind at a later stage, and you do not have to give a reason. Your doctor may also decide to stop you from being in this study if they feel it is best for you. The study may also be stopped by the study sponsor.

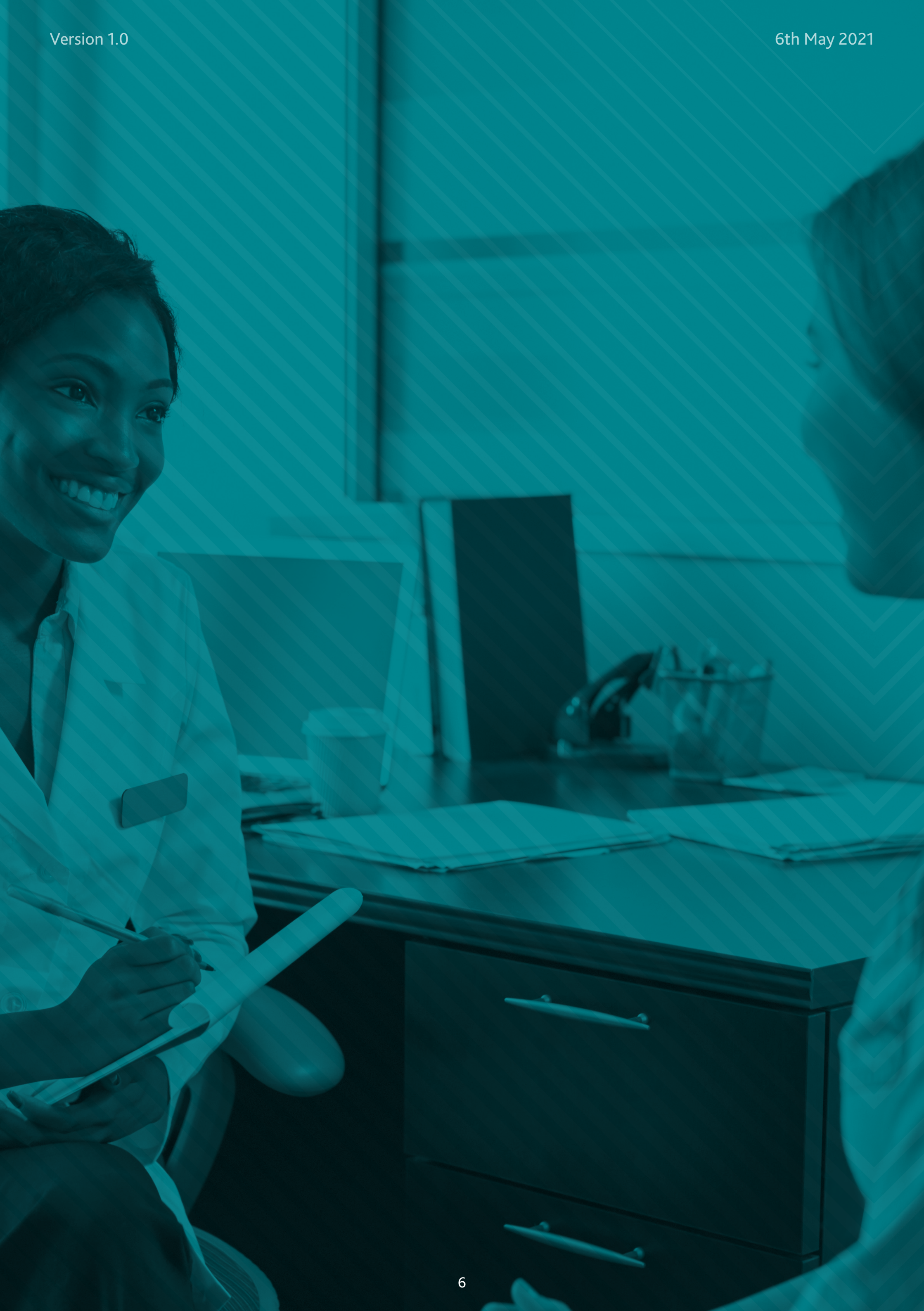

What will happen if I agree to take part?

You will be asked to attend the clinic for a number of visits:

Screening visit

If you agree to take part, a study doctor will assess you to check that you are eligible to be included in the study. This is called a screening visit. The assessment will include

- a review of your medical history
- physical examination and
- blood tests (incl. kidney, liver function, blood sugars).

Eligibility confirmed

If you are eligible to be included in the study, we will give you an appointment to come back to clinic in about 4 weeks to start the study.

Initial visit

At this visit, we will give all participants advice about changes to their diet and exercise. Half of the participants will also be randomly assigned (like flipping a coin) to get the study medication: semaglutide.

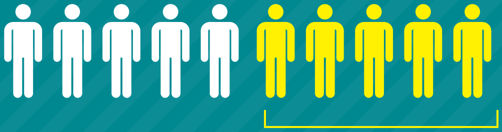

50% randomly assigned semaglutide

Treatment Stopped

If you are being treated with semaglutide during the study, treatment will be stopped after 28 weeks. Treatment will be stopped earlier if you decide to withdraw from the study or if the study doctor decides to withdraw you from the study if they feel it is best for you.

Note: If you are a woman of child-bearing potential, you must be willing to avoid pregnancy for the duration of the study and to follow your doctor's recommendations about contraception.

28 weeks | 7 clinic visits

When the study finishes, you will go back to your normal schedule of clinic visits.

Please note the following points about the study activities:

**Fasting**	You will be asked to come to study visits fasting (no food and drinks for 8 hours overnight, water is allowed).
**Semaglutide**	If you are assigned to the semaglutide treatment group, the study doctor or nurse will show you how to inject the medication into your subcutaneous tissue (the layer of tissue below your skin). You will start at a dose of 0.25 mg once a week, in order to assess tolerance to treatment. The dose of semaglutide will be increased gradually over the next 8 weeks.
**Food diaries, dietary and exercise advice**	Irrespective of your treatment group, we will give you a dietary and physical activity programme, which will help you to lose weight. At two stages during the study, we will ask you to keep a record of your meals using a 3-day food diary.
**Activity ('Actigraphy') devices**	We will give you an activity device to wear as a bracelet for 7 day periods. The device is quite simple to use, and your study doctor or nurse will talk you through how it works. We will collect it from you again at visit 5.
**Blood tests**	At various points over the course of the study, you will have blood tests to check:<br>» full blood count<br>» kidney and liver function<br>» blood sugar levels<br>» blood fat levels<br>» immune system<br>» level of HIV in the immune system cells<br>» genes responsible for your fat and sugar metabolism.
**Urine and stool samples**	We will ask you to give stool samples so we can study the bacteria in your gut. We will ask you to give urine samples so we can study for markers for inflammation, kidney and bone function.
**Pregnancy tests**	We will ask female patients to take pregnancy tests before and during the study, as pregnant women cannot be enrolled in the study. This will be either a urine or blood test.
**DXA scan**	This is a scan to assess your bone health and body composition (how much fat and muscle you have). The DXA scan will be done in the Education and Research centre in the Mater Hospital.
**Fibroscan**	This is a special kind of ultrasound scan which allows us to measure the fat in your liver and liver stiffness.

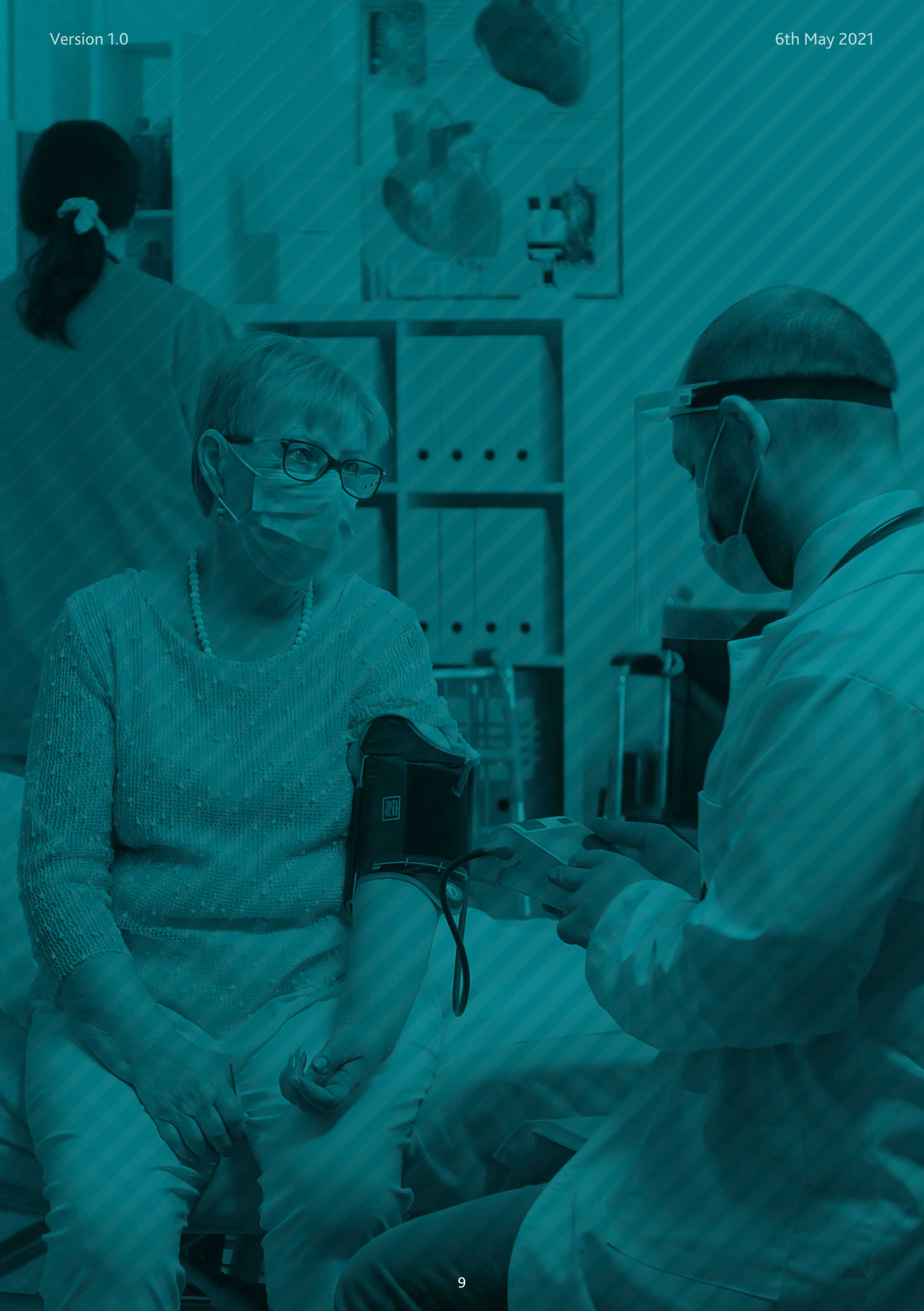

What will happen at the study visits?

Below is a diagram explaining each study visit:

**Screening** Visit	Visit 1 Week 0	Visit 2 Week 4
» Medical history and physical exam<br>» Food diary, activity monitors started<br>» Blood tests<br>» Pregnancy tests for women of child bearing potential	» Update on progress, physical exam<br>» Food diaries are collected<br>» Quality of life questionnaire<br>» Blood, urine, stool samples<br>» Subjects are randomised to receive medication + dietary/exercise advice or dietary/exercise advice alone<br>» Medication is given (to those in the semaglutide group)<br>» Activity monitor data is uploaded<br>» Pregnancy tests for women of child bearing potential<br>» DXA scan (to assess body composition and bone density)<br>» Fibroscan (to assess liver stiffness)	» Update on progress, physical exam<br>» Blood sample<br>» Medication is given (to those in the semaglutide group)<br>» Pregnancy tests for women of child bearing potential

PLEASE ATTEND APPOINTMENTS FASTING*
Please fast before each visit: no food and drinks for 8 hours (e.g. overnight). Water is allowed.

Visit 3<br>Week 8	Visit 4<br>Week 16	Visit 5<br>Week 28	**Follow-up** Visit<br>Week 40
» Update on progress, physical exam<br>» Blood, urine samples<br>» Medication is given (to those in the semaglutide group)<br>» Pregnancy tests for women of child bearing potential	» Update on progress, physical exam<br>» New food diaries started<br>» Blood, urine samples<br>» Medication is given (to those in the semaglutide group)<br>» Activity monitor data is uploaded<br>» Pregnancy tests for women of child bearing potential	» Update on progress, physical exam<br>» Food diaries are collected<br>» Quality of life questionnaire<br>» Blood, urine, stool samples<br>» Activity monitors and all unused medication are collected<br>» Pregnancy tests for women of child bearing potential<br>» DXA scan (to assess body composition and bone density)<br>» Fibroscan (to assess liver stiffness)	» Update on progress, physical exam<br>» Blood, urine, stool samples

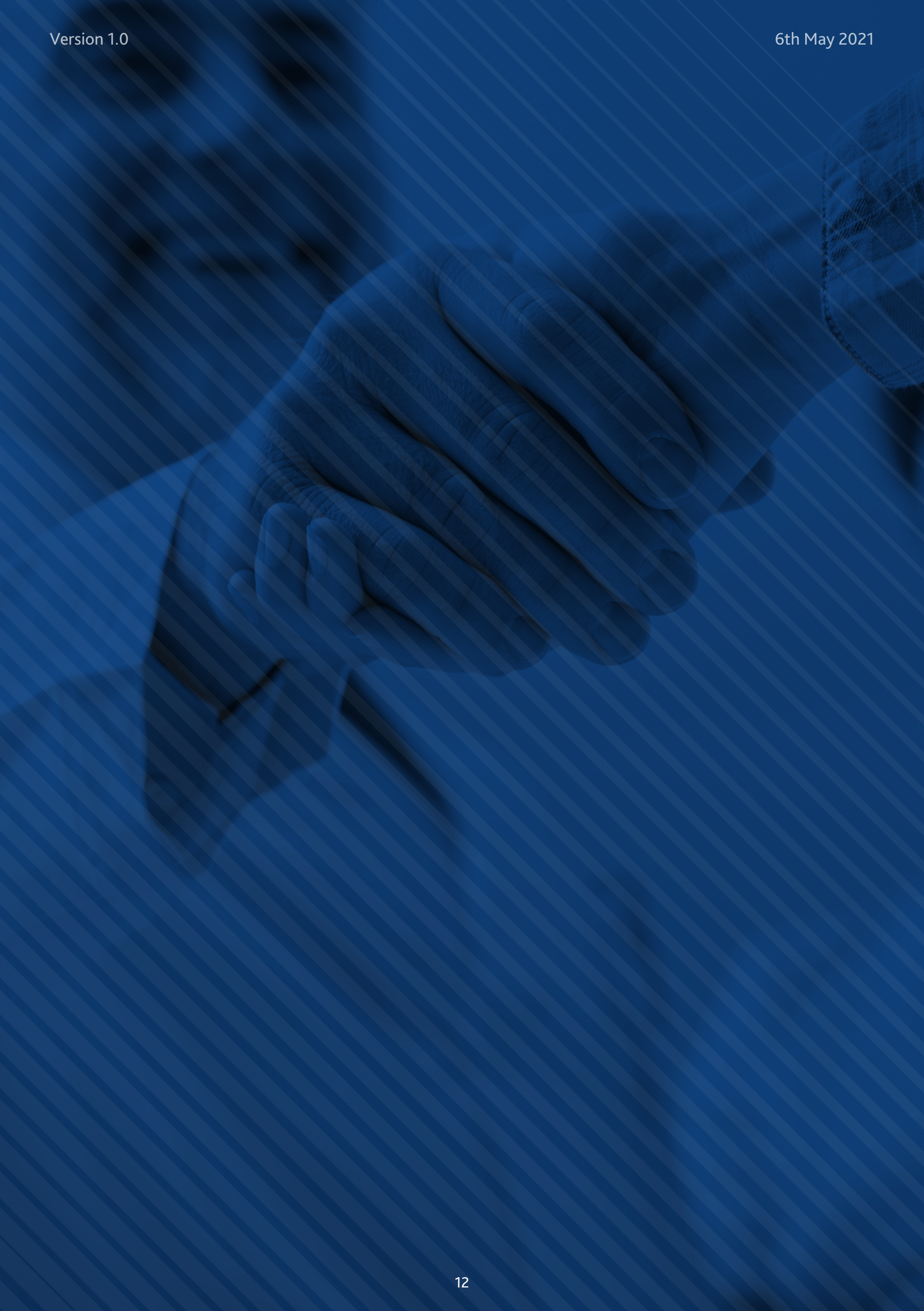

Are there any **Benefits to taking part?**

Weight loss advice

If you take part in this study, we will give you specific advice on your diet and physical activity. This may help you to lose weight. We will then actively monitor your progress.

Bone and liver health checks

We will also check your bone health with the DXA scan and your liver health with the Fibroscan. If any of the results from these tests are abnormal your doctor will take the necessary actions to address them or refer you to other specialists.

Help future patients

The results of this study may help us to understand the effect of semaglutide for people HIV and obesity. The results of this study may also help us to understand more about the immune system and the gut bacteria. This may help to improve the treatment of HIV and obesity for future patients.

Are there any Risks in taking part?

There are some small risks with this study, both related to the study medication (semaglutide) and the tests. There may also be unforeseen risks.

Semaglutide has been linked to the following side effects:

Very common	Common	Uncommon	Rare
1/10	1/100	1/1,000	1/10,000
» nausea (feeling sick)<br>» diarrhoea<br>» low blood sugar levels (when used with insulin or other diabetic medications)	» vomiting<br>» abdominal pain, bloating sensation<br>» constipation<br>» gastritis, gastro-oesophageal reflux disease<br>» gallstones<br>» fatigue<br>» increased amylase and lipase (pancreatic damage)<br>» eye complications related to diabetes<br>» loss of appetite, weight loss<br>» Feeling dizzy	» alterations in taste<br>» heart palpitations<br>» injection site reactions (rash, pain)<br>» acute pancreatitis	» allergic reactions, including anaphylaxis

Risks to pregnant women:
There may be risks to an unborn baby. You must agree to avoid pregnancy for the duration of the study and at least 2 months after finishing semaglutide.

Risks related to study procedures:

**Blood tests:**	Blood tests can be uncomfortable but rarely cause any significant problems. Rarely, people feel light-headed, faint, a blood clot forms, get bruising and/or an infection at the site of the needle stick.
**DXA scans:**	The DXA scans involve exposure to a very small amount of ionising radiation – about the same amount of radiation as a flight from Dublin to Spain. Ionising radiation can cause cell damage that may become cancerous many decades after exposure. We are all at risk of developing cancer during our lifetime. The added risk of developing cancer from having the DXA scans in this study is negligible.
**Fibroscan:**	The only risk is a mild discomfort that you might feel from the pressure of the scan probe on your abdomen and between the ribs.

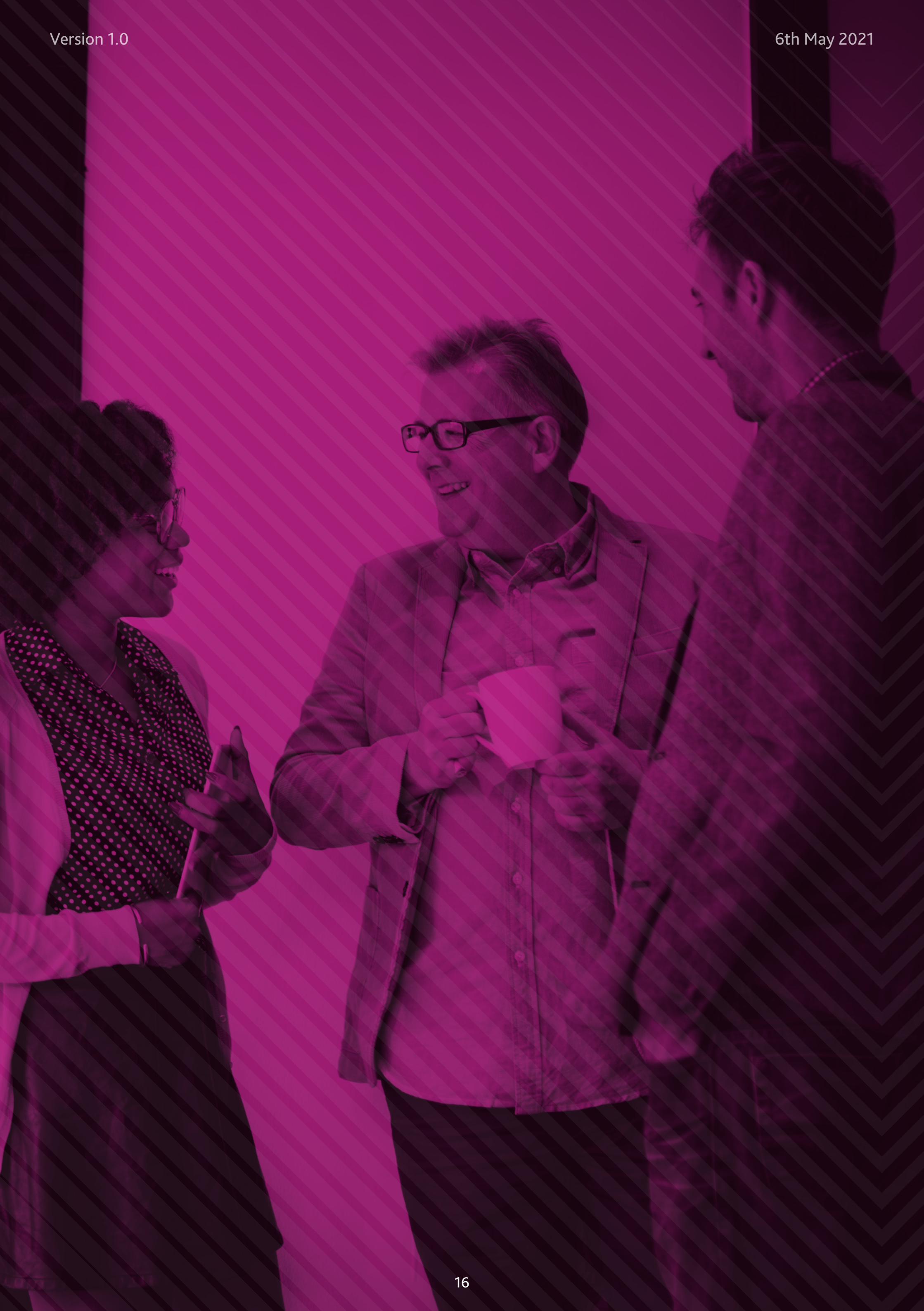

Questions you may have

Is Insurance in place for this study?

Your doctors have insurance through the Clinical Indemnity Scheme. A separate insurance policy is also in place for this clinical trial.

Who is organising and paying for this research?

This study is organised by the Centre for Experimental Pathogen Host Research (CEPHR), part of the University College Dublin (UCD) School of Medicine. This study is funded by the Irish Health Research Board (HRB).

Will I be paid for taking part in this study?

No, we are not able to pay you for taking part in this study.

Will my expenses be covered for taking part in this study?

Your travel costs for attending the clinic visits, DXA scan and Fibroscan will be covered.

Has this study been approved by an Ethics Committee?

Mater Misericordiae University Hospital and Mater Private Hospital Institutional Review Board have approved this study.

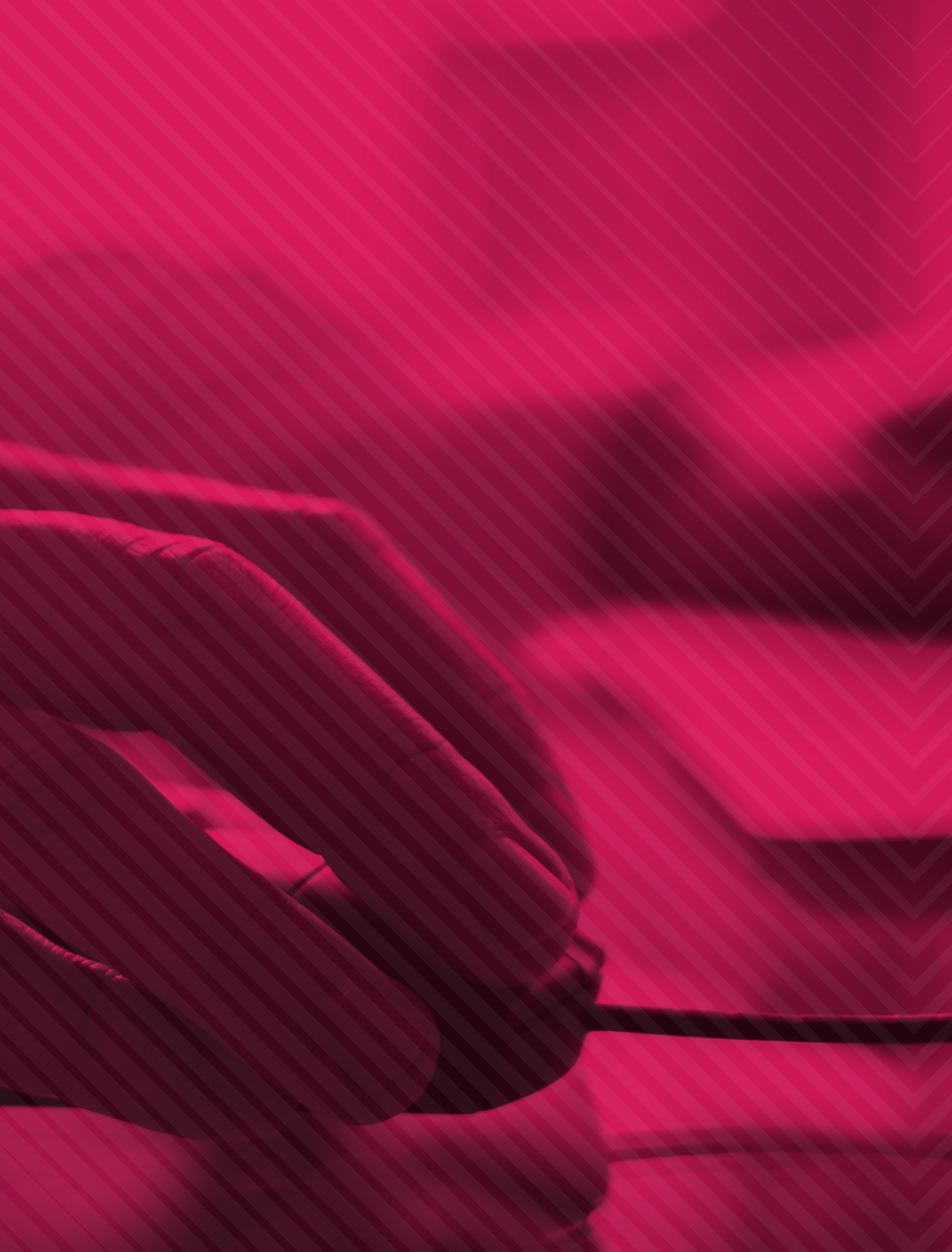

Data Protection

Will my taking part in this study be kept private?

Yes. Personal data is any information which can identify you (such as your name, address, hospital number). All records identifying you will be kept private and, to the extent permitted by the applicable laws and/or regulations, will not be made publicly available or shared without your express written consent. The study team will use a study number (a unique number which is used instead of your name) to make sure your data is kept private. Your name or other identifying details will not appear on any publications or reports from the study. To take part in this study, you must agree to share your personal data and give your consent for this data to be used and stored (this is called data processing).

Will my GP/Consultant be told that I am taking part in the study?

We will send a letter to your GP/Consultant to let them know that you are taking part in this study but only if you wish us to do so. If you don't want this information to be shared with your GP/Consultant, we will not send a letter to them.

Where can I find out more about my data protection rights?

You can find out more information on the study website: **https://swifttrial.ucd.ie** Please let your study team know if you would prefer to have a printed copy of this information.

You can also access more information on your data protection rights on the website of the Data Protection Commission, see: **https://dataprotection.ie**

Can I make a complaint about how my personal data has been used?

Yes, you can. Please see the study website to find out how to make a complaint in confidence, see: **https://swifttrial.ucd.ie**

THE MATER HOSPITAL | Ospideal Ollscoile Mater Misericordiae

Eccles Street, Dublin 7, Ireland.
Tel: +353 1 803 2000 **Fax:** +353 1 803 2404 **Email:** nmh@mater.ie **Web:** www.mater.ie

Semaglutide's Efficacy in Achieving Weight Loss for those Living with HIV

SWIFT Study CONSENT FORM

1. I, ______________________________

of ______________________________,

aged ______ years, agree to participate in the study described in the patient information statement set out in the attached form.

2. I acknowledge that I have read the patient information statement, which explains why I have been selected, the aims of the study and the nature and the possible risks of the investigation, and the statement has been explained to me to my satisfaction.

3. Before signing this consent form, I have been given the opportunity of asking any questions relating to any possible physical and mental harm I might suffer as a result of my participation and I have received satisfactory answers.

4. I give permission for researchers to look at my medical records to get information. I have been assured that information about me will be kept private and confidential.

5. I understand that I don't have to take part in this study and that I can opt out at any time. I understand that I don't have to give a reason for opting out and I understand that opting out won't affect my future medical care and my relationships with the Mater Misericordiae University Hospital or University College Dublin.

'Commitment to Excellence'

Directors: Mr. John Morgan (Chairman), Fr. Kevin Doran, Mr. Eamon Clarke, Mr. Don Mahony, Sr. Margherita Rock, Mr. Martin Cowley, Prof. Conor Keane, Ms. Mary Day, Mr. Brian Conlon, Sr. Eugene Nolan, Dr. Anthony Clarke, Mr. Kevin Murphy, Dr. Nuala Healy, Ms. Caroline Pigott

Health Services National Partnership Forum

Registered in Ireland No. 351402 **Charity No.** CHY203 **Registered Office:** Eccles Street, Dublin 7.

THE MATER HOSPITAL | UCD DUBLIN | Ospideal Ollscoile Mater Misericordiae

Eccles Street, Dublin 7, Ireland.
Tel: +353 1 803 2000 **Fax:** +353 1 803 2404 **Email:** nmh@mater.ie **Web:** www.mater.ie

6. I agree that research data gathered from the results of the study may be processed and published, provided that I cannot be identified.

7. By placing my initials in the box provided, I give permission for blood samples (including those available in routine laboratories) to be stored and/or accessed for possible future research related to the current study, for additional laboratory testing including DNA testing for changes in the genetic code of genes involved in how infections interact with my genetic makeup and/or the function of my immune system, without further consent being required, but only if the research is approved by the Institutional Review Board.

Initials: ☐

8. I understand that if I have any questions relating to my participation in this research, I may contact Principal Investigator on telephone (01) 716 5821 who will be happy to answer them.

9. I acknowledge receipt of a signed copy of this Consent Form and the Subject Information Statement. A copy will also be filed in my hospital notes and one kept on file by the investigators.

Complaints may be directed to The Patient Services Officer, MMUH at 01 803 2206.

'Commitment to Excellence'

Directors: Mr. John Morgan (Chairman), Fr. Kevin Doran, Mr. Eamon Clarke, Mr. Don Mahony, Sr. Margherita Rock, Mr. Martin Cowley, Prof. Conor Keane, Ms. Mary Day, Mr. Brian Conlon, Sr. Eugene Nolan, Dr. Anthony Clarke, Mr. Kevin Murphy, Dr. Nuala Healy, Ms. Caroline Pigott

Health Services National Partnership Forum

Registered in Ireland No. 351402 **Charity No.** CHY203 **Registered Office:** Eccles Street, Dublin 7.

THE MATER HOSPITAL
Ospideal Ollscoile Mater Misericordiae

Eccles Street, Dublin 7, Ireland.
Tel: +353 1 803 2000 **Fax:** +353 1 803 2404 **Email:** nmh@mater.ie **Web:** www.mater.ie

Participant

Signature	
PRINT name	
Date	

Person obtaining consent

Signature	
PRINT name	
Date	

Witness

Signature	
PRINT name	
Date	
Nature of Witness	

'Commitment to Excellence'

Directors: Mr. John Morgan (Chairman), Fr. Kevin Doran, Mr. Eamon Clarke, Mr. Don Mahony, Sr. Margherita Rock, Mr. Martin Cowley, Prof. Conor Keane, Ms. Mary Day, Mr. Brian Conlon, Sr. Eugene Nolan, Dr. Anthony Clarke, Mr. Kevin Murphy, Dr. Nuala Healy, Ms. Caroline Pigott

Health Services National Partnership Forum

Registered in Ireland No. 351402 **Charity No.** CHY203 **Registered Office:** Eccles Street, Dublin 7.

THE MATER HOSPITAL
Ospideal Ollscoile Mater Misericordiae
Eccles Street, Dublin 7, Ireland.
Tel: +353 1 803 2000 **Fax:** +353 1 803 2404 **Email:** nmh@mater.ie **Web:** www.mater.ie

Revocation of Consent

I hereby wish to WITHDRAW my consent to participate in the research proposal described above and understand that such withdrawal WILL NOT jeopardise any treatment or my relationship with The Mater Misericordiae University Hospital or University College Dublin.

Signature	
PRINT name	
Date	

The section for Revocation of Consent should be forwarded to:

Prof. Patrick Mallon
Catherine McAuley Education and Research Centre
Mater Misericordiae University Hospital
Nelson Street
Dublin 7

Directors: Mr. John Morgan (Chairman), Fr. Kevin Doran, Mr. Eamon Clarke, Mr. Don Mahony, Sr. Margherita Rock, Mr. Martin Cowley, Prof. Conor Keane, Ms. Mary Day, Mr. Brian Conlon, Sr. Eugene Nolan, Dr. Anthony Clarke, Mr. Kevin Murphy, Dr. Nuala Healy, Ms. Caroline Pigott

Registered in Ireland No. 351402 **Charity No.** CHY203 **Registered Office:** Eccles Street, Dublin 7.

Health Services National Partnership Forum

THE MATER HOSPITAL

Ospideal Ollscoile
Mater Misericordiae

Eccles Street, Dublin 7, Ireland.
Tel: +353 1 803 2000 **Email:** nmh@mater.ie **Web:** www.mater.ie
